# Altered synaptic structure in the hippocampus in a mouse model of Alzheimer’s disease with soluble amyloid-β oligomers and no plaque pathology

**DOI:** 10.1186/1750-1326-9-41

**Published:** 2014-10-13

**Authors:** Katherine A Price, Merina Varghese, Allison Sowa, Frank Yuk, Hannah Brautigam, Michelle E Ehrlich, Dara L Dickstein

**Affiliations:** Fishberg Department of Neuroscience, Icahn School of Medicine at Mount Sinai, One Gustave L. Levy Place, Box 1639, New York, NY 10029 USA; Friedman Brain Institute, Icahn School of Medicine at Mount Sinai, New York, NY 10029 USA; Department of Neurology, Icahn School of Medicine at Mount Sinai, New York, NY 10029 USA; Department of Pediatrics, Icahn School of Medicine at Mount Sinai, New York, NY 10029 USA

**Keywords:** Alzheimer’s disease, Aβ oligomers, Neuron, APP E693Q, Synapse, Postsynaptic density, Dendrite, Spines

## Abstract

**Background:**

Mounting evidence suggests that soluble oligomers of amyloid-β (oAβ) represent the pertinent synaptotoxic form of Aβ in sporadic Alzheimer’s disease (AD); however, the mechanistic links between oAβ and synaptic degeneration remain elusive. Most *in vivo* experiments to date have been limited to examining the toxicity of oAβ in mouse models that also possess insoluble fibrillar Aβ (fAβ), and data generated from these models can lead to ambiguous interpretations. Our goal in the present study was to examine the effects of soluble oAβ on neuronal and synaptic structure in the amyloid precursor protein (APP) E693Q (“Dutch”) mouse model of AD, which develops intraneuronal accumulation of soluble oAβ with no detectable plaques in AD-relevant brain regions. We performed quantitative analyses of neuronal pathology, including dendrite morphology, spine density, and synapse ultrastructure in individual hippocampal CA1 neurons.

**Results:**

When assessing neuronal morphology and complexity we observed significant alterations in apical but not in basal dendritic arbor length in Dutch mice compared to wild type. Moreover, Dutch mice exhibited a significant decrease in dendritic arborization with a decrease in dendritic length and number of intersections at 120 μm and 150 μm from the soma, respectively. We next examined synaptic parameters and found that while there were no differences in overall synaptic structure, Dutch mice displayed a significant reduction in the post-synaptic density (PSD) length of synapses on mushroom spines, in comparison to wild type littermates.

**Conclusion:**

The structural alterations to individual neurons in Dutch mice along with the changes in larger dendritic spines support the Aβ oligomer hypothesis, which postulates that the early cognitive impairments that occur in AD are attributed to the accumulation of soluble oAβ first affecting at the synaptic level with subsequent structural disturbances and cellular degeneration.

**Electronic supplementary material:**

The online version of this article (doi:10.1186/1750-1326-9-41) contains supplementary material, which is available to authorized users.

## Background

The neuropathological hallmarks of Alzheimer’s disease (AD) include amyloid-β (Aβ) plaque accumulation, neurofibrillary tangle formation, and synaptic and neuronal loss. How these factors ultimately contribute to memory loss and cognitive deficits that clinically characterize the disease remains unclear. In AD brains, Aβ can spontaneously aggregate and thereby assume a variety of conformational states ranging from monomers to soluble oligomers, protofibrils, and insoluble fibrils, which assemble to form extracellular plaques [1, 2]. Further characterization of this array of Aβ conformations has proven critical for deciphering which of these may be neurotoxic. The insoluble fibrillar Aβ (fAβ) found in the extracellular plaques in post-mortem AD brains has long been postulated as the initiating agent in the neurodegenerative cascade of the disease. However, many recent studies have demonstrated that the amount and distribution of amyloid plaques does not correlate with synaptic or neuronal loss, and the onset of cognitive decline in AD [3–9]. In contrast, soluble oligomeric Aβ assemblies (oAβ, also called Aβ-derived diffusible ligands), which range in size from tetramers to dodecamers and can accumulate intracellularly, show correlation with synaptic loss and synaptic impairment in many *in vitro* studies. Soluble oAβ binds preferentially to synapses [10], and addition of oAβ to mouse hippocampal slices results in inhibition of long-term potentiation [11]. This correlation has also been observed *in vivo;* the injection of oAβ directly into the hippocampus of rats resulted in deficits in learning and memory [2, 12, 13]. While these data suggest that soluble oAβ represents the neurotoxic species in AD over insoluble fibrillar forms, the relationships between oAβ, neurodegeneration, and cognitive decline remain poorly defined, with most studies having only examined the toxicity of oAβ *in vitro* or *in vivo* in mouse models that possess soluble oAβ as well insoluble fAβ, and Aβ plaques (see [13, 14] for review). Data generated from these mouse models produced results that can be difficult to interpret due to the presence of multiple Aβ conformations.

Mutations in amyloid precursor protein (*APP*) are observed in patients with familial early-onset AD or dementia caused by cerebral amyloid angiopathy. Of the *APP* mutations causing AD or cerebral amyloid angiopathy, four occur at the E693 position of the protein, the Dutch (E693Q) [15], Arctic (E693G) [16] and Italian (E693K) [17] mutations and a deletion (E693Δ) [18]. In contrast to the pathological amyloid deposition observed in AD, patients who carry the *APP* E693G (Arctic) or E693Δ variants show little or no fibrillar Aβ as detected by amyloid imaging [18, 19]. Current imaging technologies cannot detect soluble oAβ, which may be present in the brain, affect synaptic function and lead to the cognitive deficits observed in these patients. The present investigation sought to examine the effects of soluble oAβ on neuronal and synaptic structure in the APP E693Q (“Dutch”; DU) mouse model of AD that displays intraneuronal accumulation of soluble oAβ with no detectable plaques. This mouse model expresses the E693Q mutation of the *APP* [20]. Missense mutations in *APP* located near the middle of the Aβ domain influence the propensity of Aβ to form oligomeric assemblies by disrupting the salt bridges on the protein that typically stabilize parallel β-pleated sheets and favor fibril and plaque formation, thereby promoting the formation and intraneuronal accumulation of oAβ [21]. Severe meningocortical vascular deposition of Aβ in patients with hereditary cerebral hemorrhage with amyloidosis caused by the DU mutation has also been described. Interestingly, these patients consistently develop cerebral hemorrhages but rarely display significant parenchymal amyloid plaque accumulation [22, 23]. This was initially proposed to be related to the ratio of Aβ42/40, with Aβ40 being the dominant species, however subsequent studies revealed Aβ42 also plays a role in vascular amyloid formation [22, 23]. Recent work by Gandy and colleagues indicates that the level of soluble oAβ in the DU mouse model correlates with diminished performance in the water maze compared to non-transgenic wild type (WT) littermates at 12 months [20], indicating that DU mice, which do not demonstrate extracellular deposits, exhibit perturbed hippocampus-associated spatial learning and memory.

Our goal was to complement these behavioral findings by performing quantitative analyses of neuronal pathology, including dendrite morphology, as well as spine and synapse numbers in individual hippocampal CA1 neurons. These morphological features of the neuron are the site of critical memory forming processes [24–26]. Twelve month-old DU mice were assessed, along with non-transgenic WT littermates, for comparison of dendritic length and complexity, and quantification of dendritic spines and synapse densities. Structural components of synapses such as postsynaptic density (PSD) length and synapse head diameter of individual pyramidal CA1 neurons were also examined. We show that DU mice displayed morphological changes in CA1 neurons compared to WT mice, including significantly reduced dendritic arborization of apical dendrites along with decreases in PSD length of synapses on mushroom spines. These structural alterations to neurons in DU mice support the concepts that the soluble oAβ species have adverse effects at the synaptic level along with major structural disturbances, and that changes at the synapse correlate with early cognitive impairments in AD.

## Results

### Confirmation of oAβ in the DU mouse model

**Figure 1:**
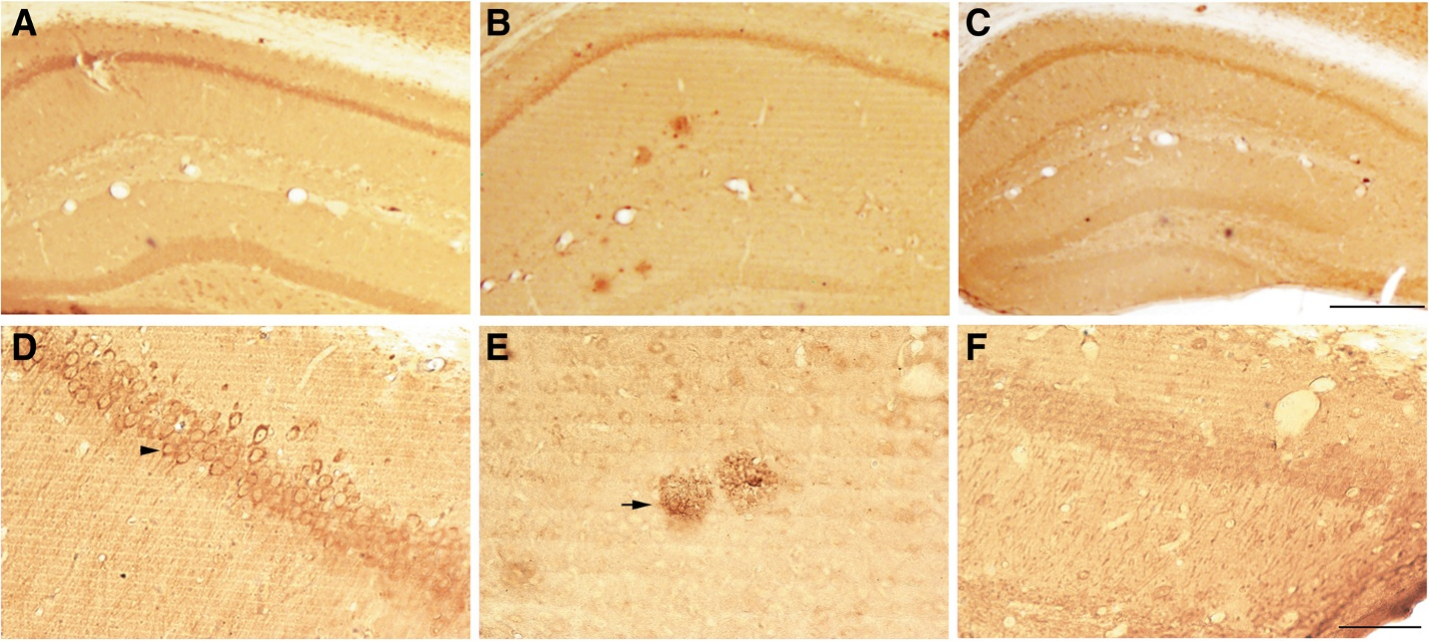
**APP/Aβ pathology in the hippocampus of Dutch mutant APP and TgCRND8 mutant APP transgenic mice.** Aβ and APP species were visualized using the 6E10 antibody. Intracellular APP/Aβ-like immunoreactivity (arrowhead) is evident in DU mice **(A and D)** while extracellular amyloid plaques (black arrow) are evident in TgCRND8 APP transgenic mice **(B and E)**. No APP/Aβ immunoreactivity is seen in WT mice **(C and F)**. **A-C** scale = 500 μm; **D-F** scale = 100 μm.

Before beginning the neuroanatomical comparisons in DU and WT mice, it was first necessary to confirm transgene expression and the presence of oAβ in the DU mice. Using the 6E10 anti-Aβ antibody we found that DU mice expressed amyloid and exhibited accumulation of intracellular Aβ (Figure 1A, D) and not extracellular plaques as in the TgCRND8 mice (Figure 1B, E), confirming results from previous studies [20, 27]. No amyloid pathology was observed in WT mice (Figure 1C, F).

### Effects of oAβ on dendritic length and arborization

**Figure 2:**
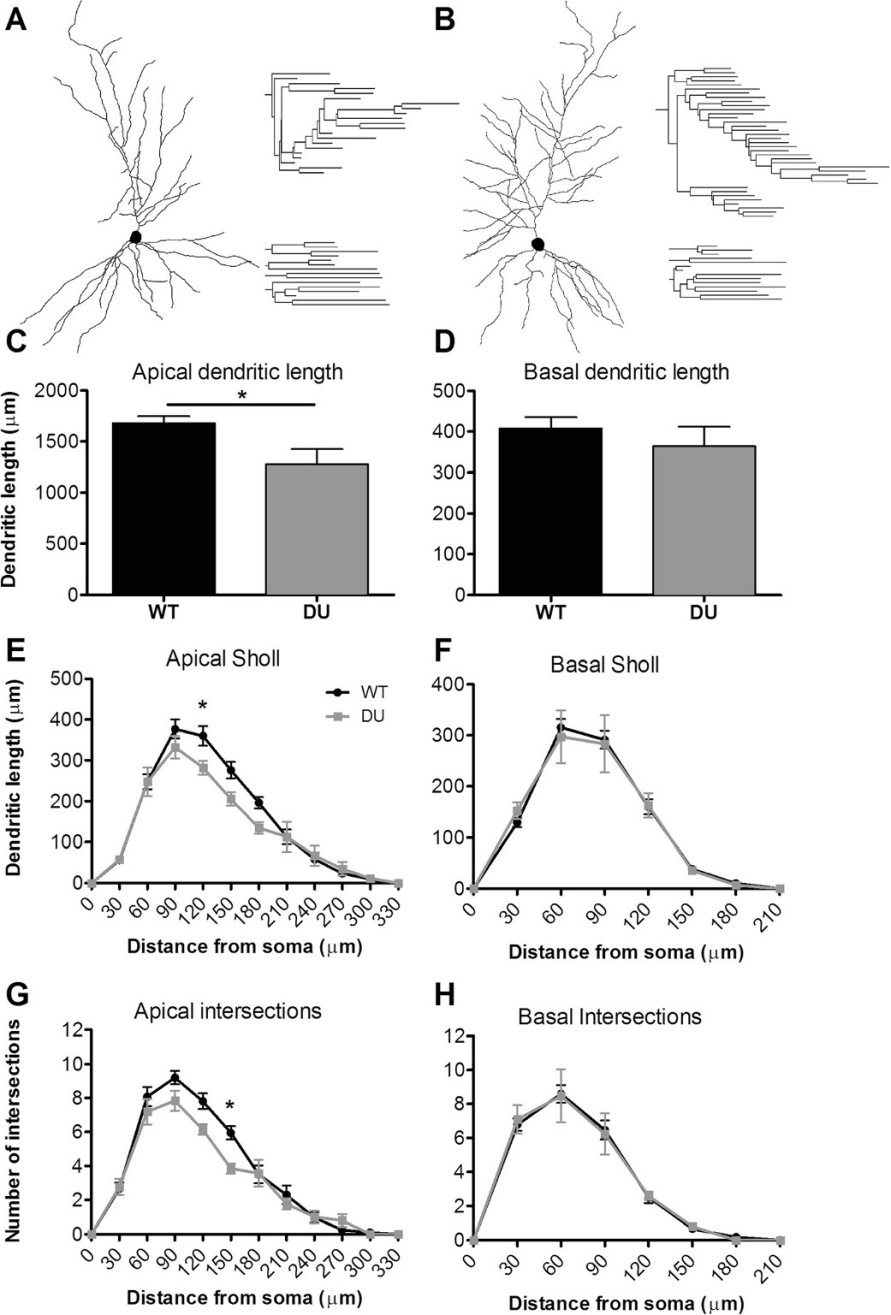
**Alterations in neuronal morphology with amyloid pathology.** Representative neuronal traces of WT and DU neurons and their apical and basal dendrograms are represented in **A and B**, respectively. Significant differences were observed in apical but not basal length **(C and D)**. Sholl analysis revealed a significant decrease in apical dendritic length **(E)** and intersections at 120 μm and 150 μm away from the soma, respectively, in DU neurons compared to WT **(G)**, but this finding was not repeated for basal dendrites **(F, H)**. Data represent group mean ± SEM (n = 11 WT and 5 DU). *p < 0.05.

We assessed whether CA1 neurons from DU mice would display differences in dendritic length and complexity compared to neurons from WT mice, and predicted that apical and basal dendrites from DU mice would be atrophic, and display perturbed arborization and complexity compared to WT mice. Representative examples of CA1 dendritic arbor reconstructions and dendrograms of WT and DU neurons are depicted in Figure 2A and B. Dendritic measurements revealed a significant decrease in the length of apical dendrites (Figure 2C, *t*_(15)_ = 2.828; p = 0.013) but not in basal dendrites (Figure 2D, *t*_(15)_ = 0.857; p = 0.405) in DU mice compared to WT (Table 1). To characterize the branching characteristics of individual neurons and their radial complexity, we performed Sholl analysis on apical and basal dendrites of DU and WT CA1 neurons. Sholl analysis of apical dendritic length and intersections revealed significant effect of genotype on the dendritic length (Figure 2E, F_(1, 168)_ = 6.31 p = 0.013) and intersections (Figure 2G, F_(1, 168)_ = 0.82, *p* = 0.01) in DU dendrites compared to WT mice. These differences were most pronounced at a distance of 120 μm and 150 μm away from the soma, respectively. When basal dendrites were analyzed there were no significant differences observed for both basal dendritic length (Figure 2F, F_(1, 112)_ = 0.01 p = 0.923) and basal intersections (Figure 2H, F_(1, 112)_ = 6.31 p = 0.999) between DU and WT mice. Together these data suggest that soluble oAβ primarily affects the apical dendritic arbors, with no effect on basal dendrites of CA1 pyramidal neurons when it accumulates *in situ*. It remains to be assessed whether these effects are the result of decreased input to the CA1 from other affected hippocampal areas or a local effect in the CA1 region.

**Table 1:** **Apical dendritic, spine and synapse morphology from CA1 pyramidal neurons of DU and WT mice**

	Genotype	
	DU	WT
**Apical dendritic length (μm)**	1277.65 ± 147.79	1507.37 ± 154.86
**Spine density (Spines/μm)**		
Total	2.676 ± 0.164	2.674 ± 0.118
Thin	1.585 ± 0.307	1.548 ± 0.133
Stubby	1.114 ± 0.177	1.007± 0.097
Mushroom	0.199 ± 0.014	0.237± 0.012
**Synapse density (μm** ^**3**^ **)**		
Total	2.897 ± 0.304	2.830 ± 0.250
Perforated	0.097 ± 0.013	0.123 ± 0.023
Non-perforated	2.800 ± 0.297	2.829 ± 0.250
**Synapse morphology (μm)**		
PSD length	0.300 ± 0.007	0.327 ± 0.006
Head Diameter	0.529 ± 0.014	0.551 ± 0.006

### Effects of oAβ on spine density and spine subtype

**Figure 3:**
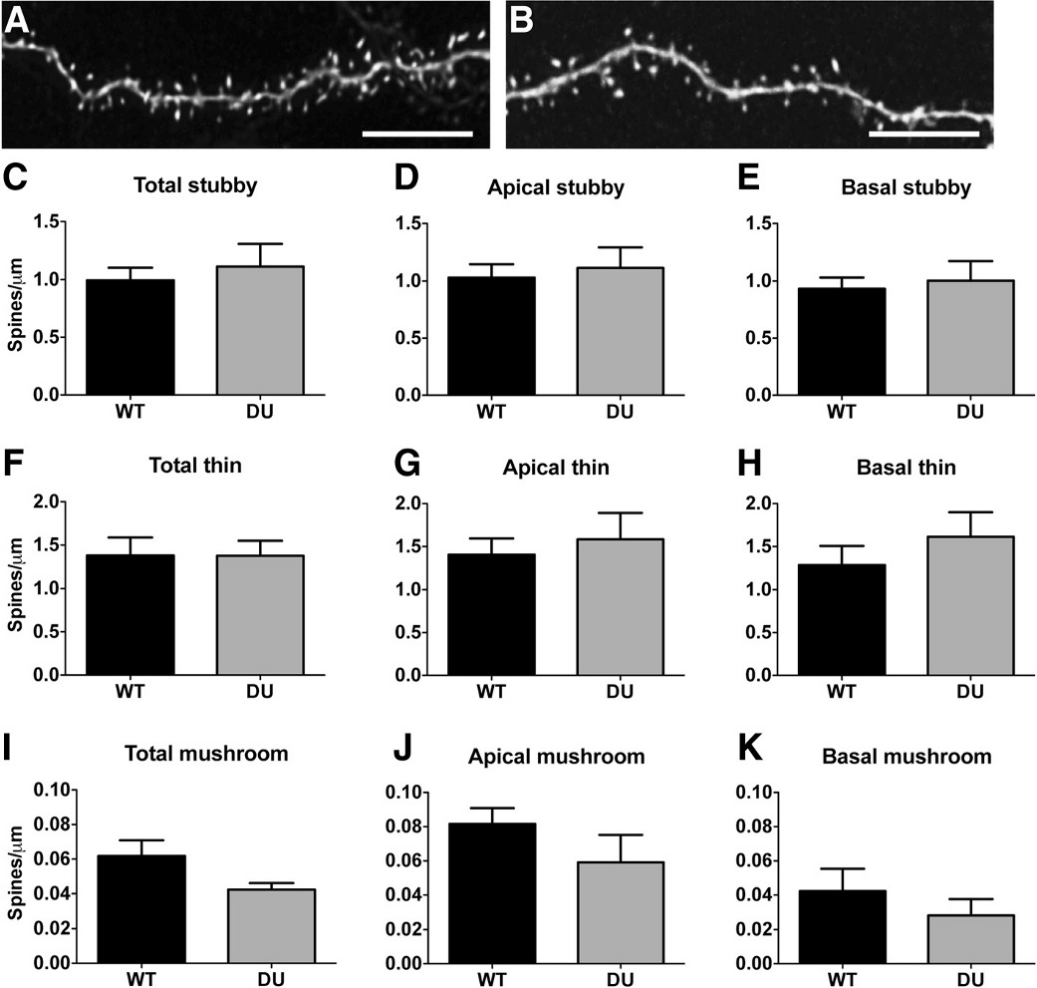
**No significant changes in dendritic spine density in DU mice compared to controls.** Representative images of deconvolved WT and DU dendrites in 3D with spine visualization **(A & B)**. When spine density was analyzed, no changes were observed for total, thin, and stubby spines on apical and basal dendrites in DU and WT neurons **(C-H)**. Analysis of mushroom spines revealed a decrease in density however, this did not reach significance **(I-K)**. Data represent group mean ± SEM. Scale bars = 5 μm.

With the dendritic atrophy observed in apical dendrites in response to the accumulation of soluble oAβ, it is also possible that there may be alterations in dendritic spine density or the proportion of spine subtypes. We therefore analyzed WT and DU neurons for changes in the density of dendritic spines. We compared overall spine density of DU and WT mice, and also examined possible changes in thin, stubby, and mushroom spines, separately (Figure 3; Table 1). We observed no significant differences in the density of total, apical or basal stubby and thin spines in DU mice compared to WT mice (Figure 3C-H). When we analyzed total mushroom spine density we found a slight decrease between Dutch mice and controls (*t*_(8)_ = 1.967; p = 0.085). When we separated spines into apical and basal dendrites we observed a decrease in apical but not basal mushroom spine density (t_(8)_ = 2.193; p = 0.06; and *t*_(8)_ = 0.032; p = 0.975 respectively); however, as above, this did not reach significance. When the apical spine densities for each subtype were separated into proximal (50–100 μm from the cell soma) and distal (>100 μm from the cell soma), no differences were observed, and these data were therefore pooled.

### Effects of oAβ on synapses

**Figure 4:**
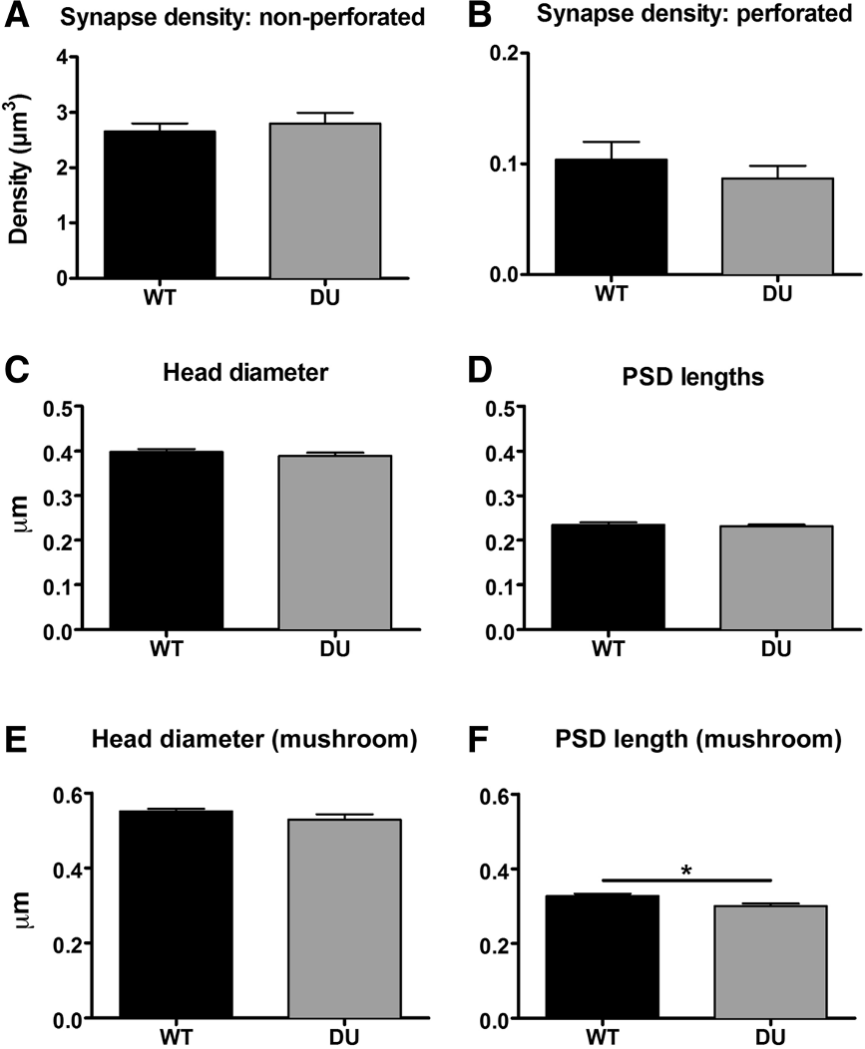
**PSD length is significantly decreased in mushroom spines in DU mice.** The synapse density of perforated and non-perforated synapses in WT and DU mice remained unchanged **(A & B)**, and the maximum head diameter of synapses and PSD lengths was also unchanged across genotypes in the SR and SLM dendritic region (**C & D**, n = 10/genotype). When synapses only on mushroom spines in the SR were analyzed, synapse head diameter remained constant, whereas a significant decrease in PSD length of DU synapses was observed (**E & F**, *p < 0.05, two-tailed *t*-test, n = 5/genotype). Data represent group mean ± SEM.

Next we asked whether oAβ affects the density of perforated and non-perforated synapses on spines from both the stratum radiatum (SR) and the stratum lacunosum-moleculare (SLM) dendritic domains. Approximately 1500 synapses were reconstructed (130/animal on average) across serial EM sections using the disector method. Analysis of the density of non-perforated and perforated synapses from SR and SLM revealed no significant difference between WT and DU mice (Figure 4A, B; Table 1). Structural alterations to individual synapses were examined by measuring the maximum head diameter and PSD lengths of synapses across DU and WT animals. When average maximum head diameter of synapses was analyzed, no major differences in mean values was observed (WT = 0.392 μm, DU = 0.393 μm, Figure 4C), and the range of values did not change significantly for each genotype (WT = 0.143 – 0.942 μm, DU = 0.182 – 0.999 μm). Similarly, we found that the means for PSD lengths for each genotype were close to identical (WT = 0.243 μm, DU = 0.238 μm, Figure 4D). When we determined the range of PSD lengths for DU and WT mice, these were also similar (WT = 0.094 – 0.609 μm, DU = 0.112 – 0.453 μm). Cumulative frequency curves of both synapse head diameter and PSD lengths to compare their distribution in DU and WT mice failed to reveal any significant differences (Kolmogorov-Smirnov test, see Additional file 1: Figure S1). When data were separated according to either SR or SLM, we also saw no changes to these synaptic parameters (data not shown).

We reasoned that there might be changes to the synaptic characteristics on specific spine subtypes. We chose to focus our investigation here on the mushroom spine subtype since this spine subtype is implicated in the formation and maintenance of memory [28, 29]. Although we did not observe any changes in maximum head diameter and PSD length in Figure 4C & D, any changes to synapses on mushroom spines may have been masked by the pooling of our synapse measurements from all spine subtypes. Therefore, we analyzed the maximum head diameter and PSD length of mushroom spines. When synapses on mushroom spines were analyzed separately in this way, we observed a significant decrease in PSD lengths for synapses on mushroom spines in DU mice compared to WT with no change in maximum head diameter (Figure 4F, *t*_(8)_ = 3.025; p = 0.016).

## Discussion

Aβ and its proposed neurotoxicity has been the focus of AD research since it was identified in the brains of AD patients three decades ago [30, 31]. The discovery that Aβ has a variety of conformations, and the lack of correlation between cognitive decline and plaque accumulation, has prompted researchers to reassess the ‘amyloid cascade hypothesis’ that dominated early AD research [32]. Recent advances in early AD diagnosis have enabled amyloid PET imaging, using ligands such as florbetapir [33] and Pittsburgh Compound-B [34] to detect Aβ deposits. However, these studies have also validated that hippocampal neurodegeneration is unrelated to rate of Aβ deposition [35]. The strong correlation between soluble conformations of Aβ and cognitive impairments, along with synaptic dysfunction and loss has shifted the focus of amyloid research towards soluble, oligomeric Aβ conformations as the ‘toxic species’ [32]. Indeed, monomeric and oligomeric forms of Aβ have been reported to accumulate in synapses of AD brains as compared to controls [36]. However, new tools and models to discriminate between different Aβ aggregation states have not been available until recently [37], and despite reports of altered dendritic spine and synaptic morphology in a variety of AD experimental models, there is limited knowledge about how soluble oAβ may affect these structural parameters in a model that does not also accumulate amyloid plaques.

In the current study, we have utilized a mouse model of AD that expresses the Dutch variant of APP. *In silico* modeling of Aβ fibrillization [38], cell-free aggregation experiments [39] and *in vitro* experiments [40, 41] showed that the Dutch mutation increased fibrillization of Aβ. Indeed, the Dutch mutation increases deposition of Aβ in vascular tissue but has the opposite effect in parenchyma of the transgenic mice, as is seen in patients carrying this mutation [42]. In contrast to the mice used in the Herzig study, which expressed both normal and Dutch APP, the DU mice we used in the current study expressed Dutch APP and only produces soluble oAβ, with no detectable plaques up to 30 months of age [20] (Figure 1). The DU mice display cognitive deficits in spatial memory tasks at 12 months of age, indicating that soluble oAβ may be inducing these effects in the absence of fAβ. Here we demonstrate that CA1 pyramidal neurons exhibit changes to neuronal morphology and total spine and synapse density. We selected the CA1 region for analysis since it is implicated in long-term contextual memory retrieval [43, 44], which is affected in AD [45]. Neocortical pyramidal neurons possess extensive apical and basal dendritic trees, which integrate information from thousands of excitatory and inhibitory synaptic inputs [6]. The morphological features of a dendrite, such as its length and complexity, can influence how a neuron processes and transmits information [46–49], and many *in vivo* studies do report neuronal atrophy with proximity to fAβ in the brain and subsequent functional deficits (see [6] for review). The effects of oAβ are less well characterized, although oAβ has been implicated in alterations to overall dendritic spine number and type in AD. Most studies on spines in AD models report either a spine loss, or a shift in the proportion of thin, stubby, or mushroom spine types, which are proposed to have unique roles, complementing their distinctive morphology [3, 50–52]. For example, thin spines contain a predominantly greater number of the *N*-methyl-D-aspartate glutamate receptors (NMDARs) compared to large, mushroom spines that contain more 2-amino-3-(3-hydroxy-5-methyl-isoxazol-4-yl)propanoic acid receptors (AMPARs), making synapses on mushroom spines functionally stronger [28]. This suggests that the more plastic, thin spines are linked to learning, whereas mushroom spines may represent more stable, ‘memory’ spines [28, 29]. A growing number of reports support the notion that spines and synapses are the initial site of Aβ-mediated neurotoxicity [24–26, 53], and it is probable that more dramatic oAβ-driven disturbances in DU mice are occurring at the synaptic receptor level, which we did not examine here. Excitatory glutamatergic NMDARs, AMPARs and other critical synaptic proteins may be affected in DU mice, with reduced expression and altered distribution of NMDA and AMPA receptors. Other *in vitro* and *ex vivo* experimental models have reported direct binding of soluble Aβ to synaptic proteins at the PSD [53–55] and these observations are supported by the decrease in PSD length in DU mice we observed in this study.

The significant decrease we observed in PSD length of synapses only on apical mushroom spines may reflect disturbances in synaptic function and memory impairment in the presence of oAβ. A similar finding was reported by Nicholson et al. (2004) in their study of aged, learning-impaired Long Evans rats. These authors reported a significant (~30%) decrease in PSD length of CA1 SR synapses in the impaired group compared to control rats [56], and noted that the decrease was only apparent in perforated synapses, which are defined by a discontinuity of the electron dense plate on the post-synaptic membrane into two or more parts [57, 58]. Although the decrease in PSD length we observed in DU mice for synapses in the same brain region were not as dramatic, our data is supported by this study as perforated synapses typically occur only on mushroom spines implicated in memory formation [28, 29]. This is because the shape of the PSD can increase in complexity with increasing spine size [57]. Furthermore, perforated synapses have been proposed to enhance synaptic efficacy by increasing the surface area for receptor activation [56, 59, 60]. A decrease in PSD length would therefore reduce glutamate receptor content and impair synaptic efficacy, leading to functional disturbances [60]. Within the context of the current study, a decrease in PSD length suggests that hippocampal perforated synapses are selectively affected by soluble oAβ, correlating with the observed cognitive deficits in 12 month-old DU mice. Alternatively, it is possible that although we did not observe any changes in perforated and non-perforated synapses or total synapse number, the DU mice may have an increased proportion of AMPA-type ‘silent’ synapses. These express NMDARs, but lack functional AMPARs [61], and do not generate a synaptic response following glutamate release at normal resting membrane potentials [62]. In electron micrographs, they are structurally identical to functional synapses, and can only be distinguished by a lack of AMPA immunoreactivity [62–67]. When a study by Geinisman et al. (2004) found no changes in perforated and non-perforated synapses, and total synapse numbers in the SR dendritic domain of CA1 neurons of cognitively impaired, aged rats compared to control rats, they argued that this may be due to an increased proportion of silent synapses [62]. Because silent synapses may become functional during postnatal development and the induction of hippocampal long-term potentiation, it is also possible that functional synapses in CA1 pyramidal neurons become increasingly silent with age [62], leading to a loss of synaptic activity without diminished synapse numbers. Further support for an oAβ-driven increase in silent synapses comes from other work using both the transgenic mice carrying the Swedish APP mutation and the external application of oAβ to rat cortical neurons, which found that oAβ altered the distribution and reduced the expression of Ca^2+^/calmodulin-dependent protein kinase II (CaMKII) [68]. CaMKII is a critical scaffolding protein highly enriched at PSDs that is required for transporting AMPARs to the synapse, and switching synapses from silent to functional through AMPA delivery [69, 70]. A reduction in the expression of CaMKII following oAβ exposure therefore provides a mechanism for increased silent synapses to occur. Interestingly, no changes in dendritic spine density were also found by another group using oAβ externally applied to rat cortical cultures compared to controls [68]. Further analysis of the DU mice using immunogold electron microscopy (EM) will help to clarify if soluble oAβ accumulated *in situ* influences the proportion of silent synapses or dramatically alters the expression and distribution of synaptic receptors in the CA1. Additional behavioral and immunogold EM analyses in older DU mice (18 and 24 month-old) will also reveal whether their cognitive deficit and neuropathology worsens with age.

## Conclusions

In the present study, we report that accumulation of soluble oAβ only in DU mice leads to discernible morphological changes to neurons, with an increase in dendritic atrophy accompanied by a decrease in PSD length that may be responsible for the cognitive impairment seen in these mice. Many *in vitro* studies on the putative neurotoxicity of oAβ to dendrites, spines, and synapses that do report changes to neuronal morphology have used synthetic oAβ preparations that may not accurately match the toxicity profile of naturally occurring oAβ species occurring *in vivo* [71]. In addition, mouse models of AD up to this point have expressed both soluble oAβ and insoluble fibrillar species, hindering clarification of the ‘conformation-specific’ neurotoxic effects of Aβ. Interestingly, in these models of AD, loss of dendritic spines or synaptic proteins has been observed prior to Aβ plaque deposition or in brain areas without Aβ plaques (reviewed in [72]). Methodological inconsistencies and the lack of adequate mouse models to assess only oAβ *in situ* prior to this point, means that care should be taken in the interpretation of individual AD studies. Importantly, this also highlights the need for consistent methodological practices across AD research for improving and accelerating our understanding of the disease. We propose that the DU mouse model of AD represents a useful tool for the assessment of oAβ-mediated synaptic changes, based on our findings of detectable synaptic changes parallel to the onset of cognitive deficits in this model. Future studies using transgenic models expressing the Dutch, Arctic or E693Δ variants of APP and aimed at the development of imaging techniques to detect oAβ, in the absence of Aβ deposits would enable correlation of these synaptic changes with a potential clinical diagnostic tool.

## Methods

### Experimental animals

Male and female 12-month old mice were used in the study. All animals were group housed in micro-isolator cages under a 12-hour light/dark cycle and given *ad libitum* access to food and water. The DU mice used in the study contain the human *APP* gene harboring the Dutch mutation (APP E693Q) under control of the *Thy1* promoter [20]. These mice exhibit intraneuronal accumulation of soluble oAβ with no detectable plaques, in addition to the appearance of amyloid-laden cerebral vessels and occasional extravasation of blood from vessels without gross hemorrhage [20]. TgCRND8 mice express double mutations within the human APP 695 isoform (Swedish K670M/N671L and Indiana V717F mutations) under control of the hamster prion promoter. These mice overexpress human Aβ by 3–4 months, have a high Aβ_42_/Aβ_40_ ratio and exhibit plaque deposition, activated microglia, neuronal loss and cognitive deficits as early as 3 months of age [73–75]. All animal procedures were conducted in accordance with the National Institute of Health Guidelines for the Care and Use of Experimental Animals and were approved by the Institutional Animal Care and Use Committee at the Icahn School of Medicine at Mount Sinai. For cell loading and EM experiments, we included n = 5 mice/group and imaged a minimum of 5 neurons/mouse with DU mice being compared to WT mice.

### Mouse perfusions

Mice were anesthetized and perfused with 1% paraformaldehyde (PFA) followed by 4% PFA with 0.125% glutaraldehyde as described previously [75, 76]. The brains were removed, hemisected, postfixed overnight, and sectioned on a Vibratome (Leica VT1000S) into 200 μm-thick sections for cell loading experiments and 250 μm for EM. All sections were stored at 4°C in phosphate buffered saline (PBS) until ready for use.

### Immunohistochemistry

Free-floating hippocampal brain slices from DU and WT mice were processed for immunohistochemistry for Aβ using the 6E10 anti-Aβ antibody (Covance) as previously described [27, 77]. The mAb 6E10 is specific for amino acids 1–16 of human Aβ, with the epitope lying within amino acids 3–8 of Aβ (EFRHDS). Brain slices from TgCRND8 mice that accumulate dense cored Aβ plaques [73], were stained in parallel as a positive control. Images were acquired on a Zeiss Axiophot 2 microscope equipped with a motorized stage and video camera system using 2.5x/0.075 N.A., Plan-Neofluar and a 40x/1.3 N.A., Plan-Neofluar oil immersion objectives.

### Intracellular dye injections

For intracellular injections, sections were incubated in 4’,6-diamidino-2-phenylindole (DAPI; Vector Labs) for 5 min to reveal the cytoarchitectural features of the pyramidal layer of CA1 of the hippocampus. The sections were then mounted on nitrocellulose paper and immersed in ice-cold 0.1 M PBS. Pyramidal neurons in the CA1 were subjected to an intracellular iontophoretic injection of 5% Lucifer Yellow (Molecular Probes) in distilled water under a direct current of 3–8 nA until dye had completely filled distal processes [75, 76, 78]. Five to 10 neurons were injected per slice and placed far enough apart to avoid overlapping of their dendritic trees. Brain sections were then mounted on gelatin-coated glass slides and cover slipped in Fluoromount G slide-mounting media (Cell Lab Beckman Coulter).

### Neuronal and dendritic reconstruction

To be included in the analysis, a loaded neuron had to satisfy the following criteria: (1) reside within the pyramidal layer of the CA1 as defined by cytoarchitectural characteristics; (2) demonstrate complete filling of dendritic tree, as evidenced by well-defined endings; and (3) demonstrate intact tertiary branches, with the exception of branches that extended beyond 50 μm in radial distance from the cell soma [75, 76, 78]. Neurons meeting these criteria were reconstructed in 3-dimensions (3D) with a 40×/1.4 N.A., Plan-Apochromat oil immersion objective on a Zeiss Axiophot 2 microscope equipped with a motorized stage, video camera system, and Neurolucida morphometry software (MBF Bioscience). Using NeuroExplorer software (MBF Bioscience) total dendritic length, number of intersections, and the amount of dendritic material per radial distance from the soma, in 30-μm increments [79] were analyzed in order to assess morphological cellular diversity and potential differences between the animal groups.

### Confocal microscopy and spine acquisition

Using an approach that precludes sampling bias of spines, CA1 dendritic segments were selected with a systematic-random method [80, 81]. Dendritic segments, 20–25 μm in length, were imaged on a Zeiss CLSM 510 microscope (Zeiss) using a 100×/1.4 N.A. Plan-Apochromat objective with a digital zoom of 3.5 and an Ar/Kr laser at an excitation wavelength of 488 nm. All confocal stacks were acquired at 512 × 512 pixel resolution with a z-step of 0.1 μm, a pinhole setting of 1 Airy unit and optimal settings for gain and offset. All confocal stacks included approximately 1 μm above and below the identified dendritic segment. On average 6 z-stacks were imaged per neuron, 3 for the apical dendritic tree and 3 for the basal dendritic trees. To be optically imaged, a dendritic segment had to satisfy the following criteria: (1) the entire segment had to fall within a depth of 50 μm; (2) dendritic segments had to be either parallel or at acute angles to the coronal surface of the section; and (3) segments could not overlap other segments that would obscure visualization of spines [80, 81]. Confocal stacks were then deconvolved using an iterative blind deconvolution algorithm (AutoDeblur version 8.0.2; MediaCybernetics).

### Spine analysis

After deconvolution, confocal stacks were analyzed with NeuronStudio [82–84] (http://research.mssm.edu/cnic/) to examine global and local morphometric characteristics of dendrites and spines such as dendritic spine densities and dendritic spine shape. This software allows for automated digitization and reconstructions of neuronal morphology in 3D from multiple confocal stacks on a spatial scale and averts the subjective errors encountered during manual tracing using a Rayburst-based spine analysis [82–84]. Spines were classified as ‘stubby’ if they had a head to neck diameter ratio less than 1.1, ‘thin’ spines were identified by a head to neck diameter ratio greater than 1.1 and a maximum head diameter less than 0.4 μm. ‘Mushroom’ spines were identified by a head to neck diameter ratio greater than 1.1 and a maximum head diameter greater than 0.4 μm [85]. Five animals per genotype, five neurons per mouse and three apical and three basal dendritic segments per neuron were analyzed with each segment manually inspected and appropriate corrections made using the NeuronStudio interface.

### Electron microscopy

Coronal sections (250 μm-thick) encompassing the CA1 region of the hippocampus were prepared for EM as reported previously [86]. Briefly, slices were cryoprotected in graded phosphate buffer/glycerol washes at 4°C, and manually microdissected to obtain blocks containing the CA1 region. The blocks were rapidly freeze-plunged into liquid propane cooled by liquid nitrogen (-190°C) in a Universal cryofixation System KF80 (Reichert-Jung) and subsequently immersed in 1.5% uranyl acetate dissolved in anhydrous methanol at -90°C for 24 hours in a cryosubstitution unit (Leica). Block temperatures were raised from -90 to -45°C in steps of 4°C/hour, washed with anhydrous methanol, and infiltrated with Lowicryl resin (Electron Microscopy Sciences) at -45°C. The resin was polymerized by exposure to ultraviolet light (360 nm) for 48 hours at -45°C followed by 24 hours at 0°C. Block faces were trimmed and ultrathin sections (90 nm) were cut with a diamond knife (Diatome) on an ultramicrotome (Reichert-Jung) and serial sections of at least 5 sections were collected on formvar/carbon-coated nickel slot grids (Electron Microscopy Sciences).

### Quantitative analyses of synapse density, PSD length, and spine head diameter

**Figure 5:**
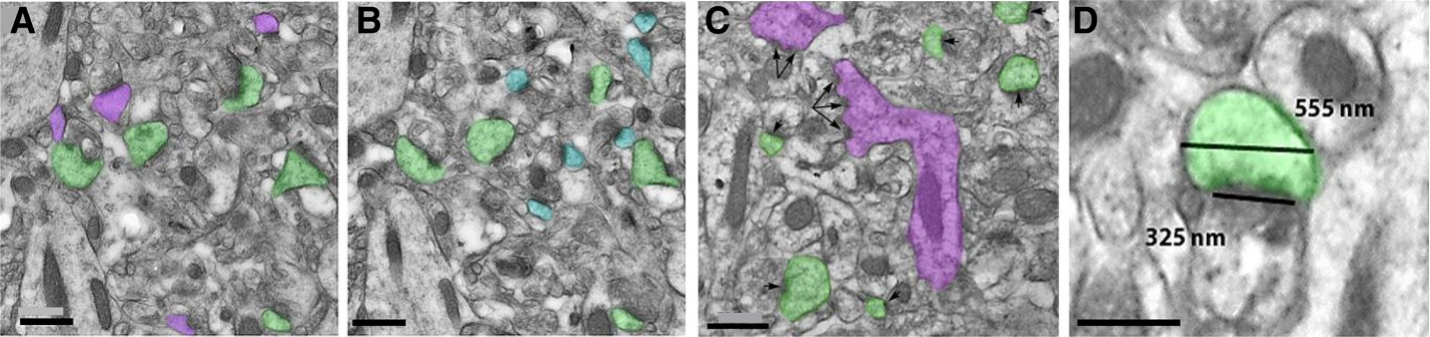
**Identification of axospinous synapses using the disector method.** Electron micrographs depicting synapses counted using the disector method **(A & B)**. Only asymmetric axospinous synapses that are present in the reference panel (purple or blue), but not in the look-up panel were counted. Synapses present in both panels (green) were not included in the analysis. Perforated synapses (**C**, purple) were classified by the presence of a discontinuity (or more than one) within the postsynaptic density (PSD) as shown by the vector arrows. Maximum head diameter of synapses and PSD length were measured as indicated in **D** (scale bars = 500 nm).

For synapse quantification, serial section micrographs were imaged at 15,000x on a Hitachi H-7000 transmission electron microscope using an AMT Advantage CCD camera (Advanced Microscopy Techniques). Twelve sets of serial images across the same set of 5 consecutive ultrathin sections were taken from the SR and SLM of the hippocampus CA1 field and imported into Adobe Photoshop (version CS5, Adobe Systems). To obtain a stereologically unbiased population of synapses for quantitative morphologic analysis, we used a disector approach on ultrathin sections as in previous reports [87, 88]. Briefly, all axospinous synapses were identified within the first and last 2 images of each 5-section serial set, and counted if they were contained in the reference image but not in the corresponding look-up image. To increase sampling efficiency, the reference image and look-up image were then reversed; thus each animal included in the current study contributed synapse density data from a total of 30 dissector pairs. The total volume examined was 11.317 μm^3^, and the height of the disector was 180 nm. Axospinous synapse density (per μm^3^) was calculated as the total number of counted synapses from both images divided by the total volume of the disector. The criteria for inclusion as an axospinous synapse included the presence of synaptic vesicles in the presynaptic terminal and a distinct asymmetric PSD separated by a clear synaptic cleft (Figure 5A & B). The synapse density was calculated as the total number of counted synapses divided by the total volume of the disectors used. For a synapse to be scored as perforated it had to display two or more separate PSD plates (Figure 5C). Spine head diameter and PSD length were of interest because they are indicative of spine plasticity and glutamate receptor representation [88]. For instance, small, thin spines are thought to be more motile and plastic than larger mushroom spines [88–90]. PSD length measurements and maximum spine head diameter were determined using a method previously described [88]. Briefly, all axospinous synapses in the middle portion of three serial sections were identified. Then, for each synapse, the longest PSD length and spine head diameter in 3 serial sections was identified and measured (Figure 5D). If the synapse only spanned 2 out of the 3 serial sections, the longest PSD and head diameter from 2 sections was measured. For the smallest class of synapses that were only present in 1 serial section, measurements were taken in that section. According to Hara et al. (2011), while this method of measuring PSD lengths and head diameter results in a 5.5% underestimation of true PSD length and head diameter, it applies across all animals and is not likely to bias systematically the outcome of group comparisons [88]. For perforated synapses, the lengths of all PSD segments were summed and the total length was used in the statistical analyses. Approximately 130 synapses were counted per animal.

### Statistics

All statistical analyses were performed using GraphPad Prism 5 software (GraphPad Software Inc). For neuron morphology and spine and synapse data, values for single cells were averaged for each animal and then per genotype and analyzed with GraphPad Prism 5. For the mean apical and basal dendritic length, and all spine and synapse data, a two-tailed Student’s *t*-test was performed. Sholl analysis was performed on apical and basal dendrites for total dendritic length, number of intersections and amount of dendritic material. Both Sholl and intersection analyses were analyzed using two‒way ANOVA followed by Bonferroni’s post‒hoc tests. To determine if the frequencies of different sized dendritic synapses differed between DU and WT mice, cumulative frequency curves of maximum spine head diameter and PSD length were examined using the Kolmogorov-Smirnov test. There were no statistical differences between male and female mice for these data, therefore sexes were grouped together. The statistical significance level for all data was set at an α level of 0.05 and all data were represented as mean ± SEM.

## Electronic supplementary material

Additional file 1: Figure S1: Cumulative frequency curves of synapse head diameter and PSD length. Cumulative frequency curves of synapse head diameter **(A)** and PSD length **(B)** of synapses on CA1 hippocampal neurons in DU and WT mice. No shift in the distribution of head diameter or PSD length of CA1 synapses was observed in DU and WT mice. (TIFF 9 MB)

## Abbreviations

Aβ: Amyloid beta peptide
AMPAR: 2-amino-3-(3-hydroxy-5-methyl-isoxazol-4-yl)propanoic acid receptor
AD: Alzheimer’s disease
APP: Amyloid precursor protein
CAMKII: Ca^2+^/calmodulin dependent protein kinase II
DAPI: 4‘,6-diamidino-2-phenylindole
DU: Dutch
EM: Electron microscopy
fAβ: Fibrillar Aβ
NMDAR: *N*-methyl-D-aspartate receptor
oAβ: Oligomeric Aβ
PBS: Phosphate-buffered saline
PFA: Paraformaldehyde
PSD: Postsynaptic density
SLM: Stratum lacunosum-moleculare
SR: Stratum radiatum
WT: Wild type.
