# Supplementary figures and images for: Altered synaptic structure in the hippocampus in a mouse model of Alzheimer’s disease with soluble amyloid-β oligomers and no plaque pathology
Source: Mol Neurodegener. 2014 Oct 13;9:41. doi: 10.1186/1750-1326-9-41 (PMC4210526; doi:10.1186/1750-1326-9-41)

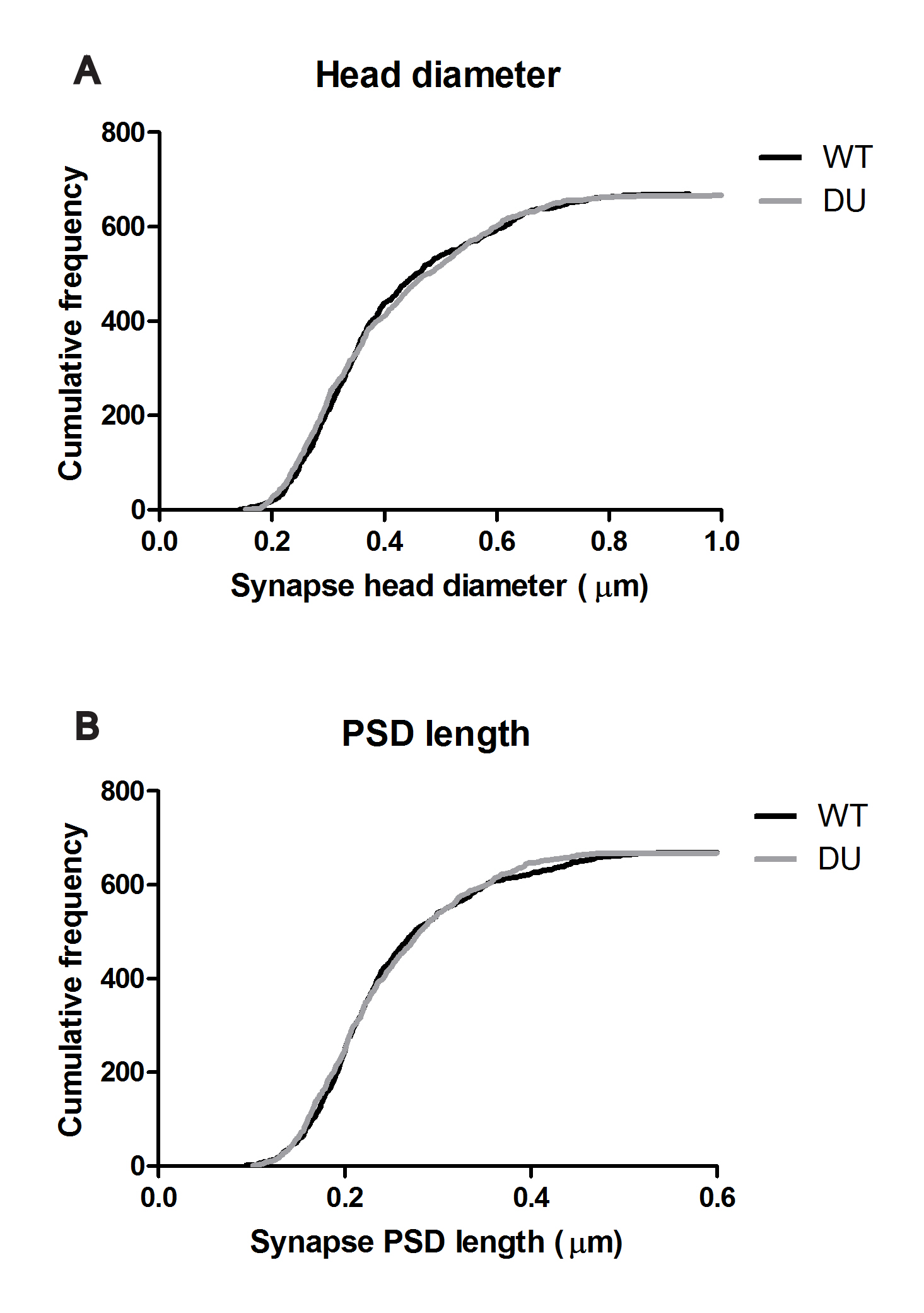

Supplement: Supplementary file 1 — Additional file 1: Figure S1: Cumulative frequency curves of synapse head diameter and PSD length. Cumulative frequency curves of synapse head diameter (A) and PSD length (B) of synapses on CA1 hippocampal neurons in DU and WT mice. No shift in the distribution of head diameter or PSD length of CA1 synapses was observed in DU and WT mice. (TIFF 9 MB) [file 13024_2014_550_MOESM1_ESM.tiff]
